# A Routing Path Construction Method for Key Dissemination Messages in Sensor Networks

**DOI:** 10.1155/2014/185156

**Published:** 2014-07-17

**Authors:** Soo Young Moon, Tae Ho Cho

**Affiliations:** College of Information and Communication Engineering, Sungkyunkwan University, Suwon 440-746, Republic of Korea

## Abstract

Authentication is an important security mechanism for detecting forged messages in a sensor network. Each cluster head (CH) in dynamic key distribution schemes forwards a key dissemination message that contains encrypted authentication keys within its cluster to next-hop nodes for the purpose of authentication. The forwarding path of the key dissemination message strongly affects the number of nodes to which the authentication keys in the message are actually distributed. We propose a routing method for the key dissemination messages to increase the number of nodes that obtain the authentication keys. In the proposed method, each node selects next-hop nodes to which the key dissemination message will be forwarded based on secret key indexes, the distance to the sink node, and the energy consumption of its neighbor nodes. The experimental results show that the proposed method can increase by 50–70% the number of nodes to which authentication keys in each cluster are distributed compared to geographic and energy-aware routing (GEAR). In addition, the proposed method can detect false reports earlier by using the distributed authentication keys, and it consumes less energy than GEAR when the false traffic ratio (FTR) is ≥10%.

## 1. Introduction

Sensor networks are large-scale computing systems that collect the data via sensors distributed in the real world. Sensor networks are composed of lightweight sensor nodes and at least one sink node. They operate autonomously for a long period of time. Sensor networks can be used for applications including battlefield reconnaissance, infrastructure management, and smart home technology [1–5].

Sensor nodes are highly resource constrained and they are prone to security threats including masquerade, message replay, message modification, and denial of service (DoS) attacks [6–8]. Authentication ensures that the identity of a communicating entity is in fact what it claims to be [8]. In authentication schemes [9–17], the authentication keys must be shared among communicating nodes. This distribution can occur in two ways: (1) static key distribution and (2) dynamic key distribution. We focus on the dynamic key distribution where each node encrypts its authentication key and disseminates it throughout the network. Since authentication keys are encrypted before dissemination, a receiving node receiving the authentication key can actually decrypt the key only when there is a corresponding decryption key in its memory. Hence, the forwarding path of the key dissemination message affects the number of nodes to which the authentication keys are distributed.

The topology and routing path changes in WSNs occur frequently due to addition and deletion of nodes. Therefore, we need to distribute authentication keys in each cluster to many nodes for detecting and dropping false reports injected through compromised nodes.

We propose a path construction method for key dissemination messages with the aim of increasing the number of nodes to which the authentication keys are actually distributed. Each node in the proposed method maintains the secret key indexes of its neighbor nodes. It selects the next forwarding node(s) of the key dissemination message based on (1) the number of authentication keys that can be distributed to the neighbors, (2) the distance from the neighbors to the sink node, and (3) the energy consumption of the neighbors. The proposed method can increase the number of nodes to which authentication keys are distributed, detect the false reports early, and reduce energy consumption. As a result, the proposed system can prolong the network's lifetime.

The contributions of our paper are as follows:

- development of a new path construction method for key dissemination messages in order to increase the number of nodes to which authentication keys in each cluster are actually distributed;
- presentation of a new filtering method that exploits the proposed path construction method to detect false reports early and reduce the energy consumed by the nodes in the network.

## 2. Related Works

Studies on route construction methods have been conducted with the aim of improving the energy efficiency of various authentication schemes. Key index-based routing (KIBR) [16] was proposed to reduce the energy consumption of false report filtering schemes. In KIBR, each node maintains the authentication key indexes of its candidate parent nodes (CPNs). A node receiving an event report forwards the report to one of the CPNs, which can verify one of the message authentication codes (MACs) in the report by considering the authentication key indexes contained in both the report and the CPNs.

KIBR can increase the filtering capability of authentication schemes and therefore conserve energy under false report attacks. That is, existing authentication schemes combined with KIBR can detect false reports earlier than can those combined with the shortest path routing. Actually, KIBR is a special case (*α*_*P*_ = 1) of the proposed route construction method. The proposed method considers not only the key index information of the CPNs but also their energy consumption, to achieve balanced energy consumption of sensor nodes. We will describe the proposed method in detail in (Section 4 *—*Proposed Method).

The path renewal method (PARM) [17] was proposed to enable balanced energy consumption among nodes and energy efficiency of the filtering schemes. Each node on the routing tree in PARM maintains the information of its parent node and child nodes. If the remaining energy of one node decreases below some threshold value, the node sends an* eviction message* to one of its child nodes. The node receiving the* eviction message* changes its parent node based on an evaluation function. The energy consumption and the key partition information of each CPN determine the output value of the function. The receiving node chooses its new parent node with the highest value of the evaluation function. Similar to KIBR, PARM can be exploited to enhance the filtering capability of an existing authentication scheme, such as statistical en route filtering (SEF) [9] and to increase the network lifetime through balanced energy consumption among nodes.

PARM can also be applied to route construction for key dissemination messages. However, each node considers in PARM the key partition information of its CPNs, which is less specific information than the key index information. Therefore, the proposed method can distribute authentication keys in each cluster to more nodes on average than PARM.

One of main assumptions in the proposed method is that each node is able to obtain its location information and its distance to the sink node. The most direct solution, loading* GPS* modules into each node, is impractical for many reasons such as production cost and limited energy of sensor nodes [18]. Therefore, many schemes [18–20] for localizing sensor nodes have been proposed by researchers.

In [18], the authors proposed a technique called AHLoS (ad hoc localization system). AHLoS only requires a limited fraction of the nodes to know their exact locations. Specifically, AHLoS enables nodes to dynamically discover their own location through a two-phase process: (1) a ranging phase and (2) an estimation phase. In the ranging phase, each node estimates its distance from its neighbors by using ranging techniques based on received RF signal strength or time of arrival (ToA) of radio frequency (RF) and ultrasonic signals. In the estimation phase, nodes use the ranging information and known location of neighboring beacon nodes to estimate their positions.

In [19], the authors proposed a location support system for in-building, mobile, location-dependent applications. It allows applications running on mobile and static nodes to learn their physical location by using “*listeners*” that hear and analyze information from* beacons*. They use beacons with combined RF and ultrasound signals in decentralized, uncoordinated architecture.

In [20], the authors proposed a GPS-less algorithm for node-position estimation. By using the algorithm, we can compute the locations of nodes by exploiting beacon nodes with known position. The algorithm reduces the position estimation errors based on the supposed position of the nodes and the distances from the beacon nodes. They implemented the algorithm on typical sensor nodes with limited resources, such as limited energy, computation speed, and memory.

## 3. Background

### 3.1. Dynamic En Route Filtering Scheme

The dynamic en route filtering (DEF) scheme [11] was proposed to efficiently detect forged reports in a network in which the topology changes dynamically. The main characteristic of DEF is that each node updates its own authentication key on a regular basis and disseminates the new key to other nodes. Hence it maintains the ability to detect false reports in a network where addition and deletion of nodes occur frequently.

There are three phases in DEF: (1) the predeployment phase, (2) the postdeployment phase, and (3) the filtering phase. In the predeployment phase, (*l* + 1) secret keys and a single seed key are assigned to each node. *l* is a system parameter that is fixed at the node deployment phase. The secret keys are randomly selected from a global key pool (GKP). Every node can construct its authentication key chain from the seed key based on the hash function. It can update its current authentication key based on the authentication key chain.

In the postdeployment phase, the nodes update their authentication keys and send the new keys to their associated cluster heads (CHs). Each authentication key is encrypted using the secret keys of the corresponding node before it is sent to the CH. Each CH organizes a key dissemination message and forwards it to *q* next-hop nodes. A node receiving the key dissemination message compares the indexes of the secret keys to encrypt the authentication keys and the indexes of its own secret keys. If there is a matching secret key, the node can decrypt the corresponding authentication key and store the key in its memory.

**Figure 1:**
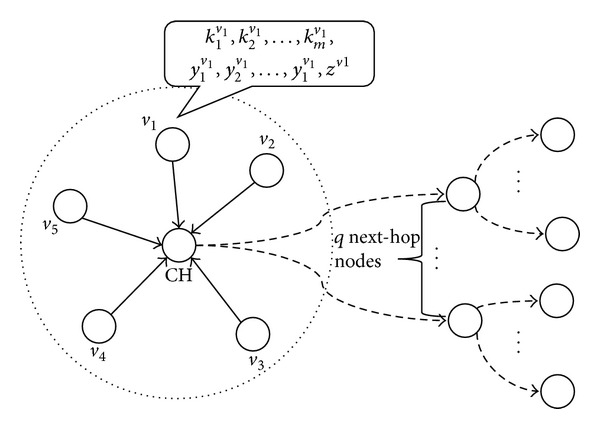
Postdeployment phase of DEF.

In the filtering phase, sensing nodes update their authentication keys, generate sensing reports, and send them to the associated CHs. Each sensing report contains a MAC generated using the new authentication key of the sending node. The CH organizes the final report and forwards it to the next-hop nodes. In addition, the CH sends a new key dissemination message that contains the authentication keys used for endorsing the final report to the next nodes. The nodes on the forwarding path verify the final report based on the authentication keys they have just obtained from the new key dissemination message. If the verification fails, they drop the report.  Figure 1 illustrates the postdeployment phase of DEF.

The circle with dotted lines represents a cluster of nodes. Each node stores an authentication key chain and (*l* + 1) secret keys (*l y*-keys and one* z*-key). Cluster member nodes (*v*_1_ − *v*_5_) encrypt their new authentication keys and send them to the CH. The CH generates a key dissemination message that contains the encrypted authentication keys and forwards it to the *q* next-hop nodes. Equation (1) shows the format of the key dissemination message:

(1) $$\begin{array}{c} v_{1},j_{1},\text{id}\left(y_{1}^{v_{1}}\right),\text{id}\left(y_{2}^{v_{1}}\right),\ldots ,\text{id}\left(y_{l}^{v_{1}}\right),\text{id}\left(z^{v_{1}}\right),\\ {\left\{k_{j_{1}}^{v_{1}}\right\}}_{{y_{1}}^{v_{1}}},{\left\{k_{j_{1}}^{v_{1}}\right\}}_{{y_{2}}^{v_{1}}},\ldots ,{\left\{k_{j_{1}}^{v_{1}}\right\}}_{{y_{l}}^{v_{1}}},{\left\{k_{j_{1}}^{v_{1}}\right\}}_{z^{v_{1}}}\\ v_{2},j_{2},\text{id}\left(y_{1}^{v_{2}}\right),\text{id}\left(y_{2}^{v_{2}}\right),\ldots ,\text{id}\left(y_{1}^{v_{2}}\right),\text{id}\left(z^{v_{2}}\right),\\ {\left\{k_{j_{2}}^{v_{2}}\right\}}_{{y_{1}}^{v_{2}}},{\left\{k_{j_{2}}^{v_{2}}\right\}}_{{y_{2}}^{v_{2}}},\ldots ,{\left\{k_{j_{2}}^{v_{2}}\right\}}_{{y_{l}}^{v_{2}}},{\left\{k_{j_{2}}^{v_{2}}\right\}}_{z^{v_{2}}}\\ \vdots \\ v_{5},j_{5},\text{id}\left(y_{1}^{v_{5}}\right),\text{id}\left(y_{2}^{v_{5}}\right),\ldots ,\text{id}\left(y_{1}^{v_{5}}\right),\text{id}\left(z^{v_{5}}\right),\\ {\left\{k_{j_{5}}^{v_{5}}\right\}}_{{y_{1}}^{v_{5}}},{\left\{k_{j_{5}}^{v_{5}}\right\}}_{{y_{2}}^{v_{5}}},\ldots ,{\left\{k_{j_{5}}^{v_{5}}\right\}}_{{y_{l}}^{v_{5}}},{\left\{k_{j_{5}}^{v_{5}}\right\}}_{z^{v_{5}}}. \end{array}$$

Each key dissemination message contains the cluster member ID (*v*_*i*_), authentication key index (*j*_*i*_), and the* y*-key and* z*-key indexes (id(*y*_1_^*vi*^), …, id(*y*_*l*_^*vi*^), id(*z* ^*v*1^)) of each node in the cluster. A node receiving the key dissemination message compares the* y*-key and* z*-key indexes in the message with those of its own keys. If there is a matching key, it decrypts the corresponding authentication keys and stores them in its memory. It then forwards the message to the *q* next-hop nodes based on the underlying routing protocol.

### 3.2. Geographical and Energy-Aware Routing (GEAR)

Geographical and energy-aware routing (GEAR) [21] is a routing protocol that considers the distance to the destination and energy consumption of neighbor nodes at each node when choosing the next node on the forwarding path. The objective of GEAR is to achieve balanced energy consumption among nodes and improve overall energy efficiency, leading to an increase in network lifetime. Each node in GEAR selects the next node on the message forwarding path based on the* learned cost* as shown in the following equation:

(2) $$\begin{array}{c} h\left(N,R\right)=h\left(N_{\min},R\right)+C\left(N,N_{\min}\right). \end{array}$$

In (2), *h*(*N*, *R*) is the* learned cost* from the node *N* to the destination *R* of the message. It is determined as the sum of the least* learned cost h*(*N*_min⁡_, *R*) of its neighbors and the link cost *C*(*N*, *N*_min⁡_). The following equation shows the derivation of the* estimated cost* from the node *N* to *R*, which is the default value for *h*(*N*, *R*):

(3) $$\begin{array}{c} c\left(N,R\right)=\alpha_{G}\cdot d\left(N,R\right)+\left(1-\alpha_{G}\right)\cdot e\left(N\right). \end{array}$$

In (3), *α*_*G*_ and (1 − *α*_*G*_) are weight values for the two factors: the distance from the node *N* to the destination *R* and the energy consumption of the node *N*, respectively. GEAR can deliver 25–35% more packets in a uniform traffic environment than can greedy perimeter stateless routing (GPSR) [22], which is a geographic routing protocol.

## 4. Proposed Method

### 4.1. Operation

**Figure 2:**
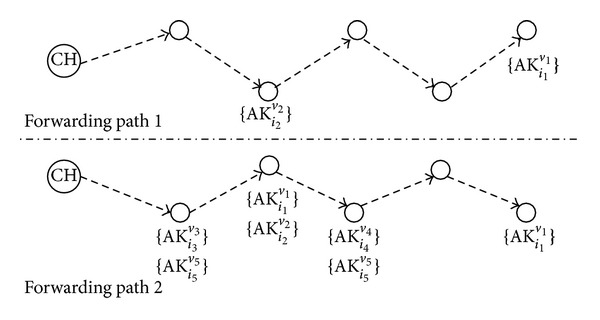
Effects of the forwarding path of a key dissemination message on key dissemination.

When a node in DEF forwards a key dissemination message, it selects *q* next-hop nodes based on the distance or hop counts from each neighbor to the sink node, the link quality from the neighbor to the sink node, and the energy consumption of the neighbor. A node receiving the key dissemination message can obtain one of the authentication keys from the message only when it can decrypt the authentication key by using one of its secret keys. Hence, the forwarding path of the key dissemination message strongly affects the number of nodes to which the authentication keys in the message are actually distributed, as shown in Figure 2.

In Figure 2, the number of nodes that obtain at least one authentication key from the key dissemination message on forwarding path 2 is larger than the number on forwarding path 1 because of the distribution of secret keys belonging to the nodes on the paths. We can increase the number of nodes that obtain at least one authentication key from the key dissemination message by selecting, at each node, the *q* next-hop nodes with consideration for the secret key indexes of the neighbor nodes. We will discuss this further in Section 5.

There are several assumptions in the proposed method, as follows. Each node deployed in the network field is aware of the distance between itself and the sink node. The density of the nodes is sufficient such that each node is associated with at least one candidate parent node (CPN), which is one that can be selected as the next node to which a key dissemination message or an event report will be forwarded. Sensor nodes after deployment organize themselves into several clusters autonomously. We can exploit any clustering methods such as PebbleNet [23] and LEACH [24].

We assume that the sensor field is divided into a geographic grid and sensor nodes within the same cell organize a cluster. Each cluster is composed of one CH and a few member nodes. The member nodes send their new authentication keys and sensing reports to their CH, and the CH organizes and forwards to the next node a key dissemination message or a final event report. The sink node manages a GKP which is a set of secret keys shared by the nodes in the network. Each node generates its own authentication key chain from its seed key based on a hash function. It periodically updates its current authentication key based on the key chain. The length of the authentication key chain is sufficiently long such that each node can repetitively update its authentication key during its lifetime.

**Figure 3:**
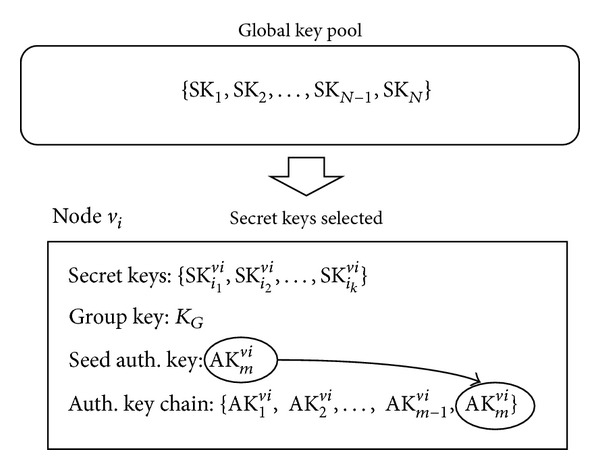
Predistribution of a seed authentication key and secret keys.

In the predeployment phase, a seed authentication key to authenticate/verify event reports and secret keys to encrypt/decrypt authentication keys are distributed to each node.  Figure 3 illustrates the predistribution of the keys in the predeployment phase.

The GKP is composed of *N* secret keys and we assign *k* secret keys, which are randomly selected from the GKP, to each node before deployment. Then, the probability of any two nodes sharing at least one secret key is as follows [11]:

(4) $$\begin{array}{c} p=1-\frac{\left(\begin{array}{c} N-k\\ k \end{array}\right)}{\left(\begin{array}{c} N\\ k \end{array}\right)}, \end{array}$$

where *k* is the number of secret keys loaded onto each node and is limited by the memory size of each node. For example, if the size of each key is 64 bits and *k* = 50, the memory overhead becomes 400 bytes.

For a given *k*, *k*/*N* should not be too small because the ratio affects the probability *p* that any two nodes share at least one secret key. For example, if *k* = 50 and *N* = 1000, then *p* ≈ 0.93. In another case, however, if *k* = 50 and *N* = 10000, then *p* ≈ 0.22, which is too low.

Every node can construct its own authentication key chain of length *m* from the seed key based on a hash function *h*  (*h*(AK_*i*_) = AK_*i*−1_). The node may store the entire authentication key chain in its memory, but it is also possible that it may store only one key for a given time and periodically update the key in order to minimize memory overhead. When we do not store the authentication key chain in the node, the node only stores the seed key and the hash function and generates new authentication key whenever it is necessary. That is, the new authentication key AK_*i*_ can be computed by performing (*m* − *i*) hash computations (AK_*i*_ = *h* ^(*m*−*i*)^AK_*m*_). The approach will increase computation overhead for authentication key update but will reduce memory overhead for storing the whole key chain. The orders of authentication key generation and authentication key usage are opposite. Hence, a node initially uses the authentication key AK_1_  and sequentially updates the authentication keyto AK_2_, AK_3_, …, AK_*m*_. This mechanism provides “forward secrecy,” which means that even if an attacker comes to know an authentication key, the next authentication key to be used cannot be predicted because of the one-way property of the hash chain. An authentication key of each node is updated in every single round that is defined as a period between two consecutive cluster reorganizations due to addition and deletion of nodes. We assume that a round is composed of 100 events. We also assume that the length of each authentication key chain is sufficiently long to enable periodic update of an authentication key of a node's lifetime. Additionally, a group key *K*_*G*_ is assigned to each node to provide authenticity to control messages. If a node in the network is compromised, the group key should be updated and distributed to the remaining nodes. Secure and efficient method to update and distribute the group key is out of scope of the paper.

In the neighbor discovery phase, each node broadcasts a* Hello* message within its transmission range. The* Hello* message contains the sender's ID, the distance from the sender and the sink node, the secret key indexes of the sender, and a MAC. The MAC in the* Hello* message is generated using the group key *K*_*G*_ and it is exploited to detect a forged* Hello* message.

**Figure 4:**
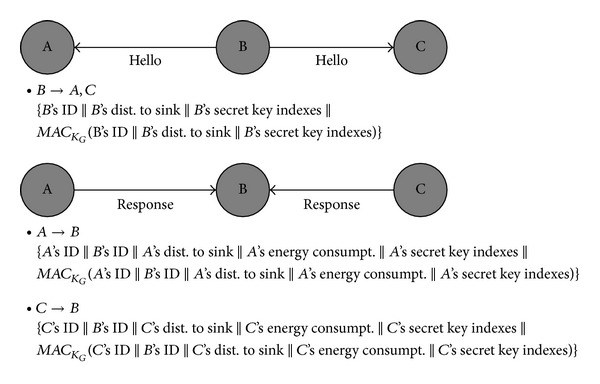
Neighbor discovery process using Hello and Response messages.

The node receiving the* Hello* message verifies the message, and if the verification result is true, it replies to the sender with a* Response* message. The* Response* message includes the receiver's ID, the distance from the receiver to the sink node, the energy consumption of the receiver, the secret key indexes of the receiver, and a MAC. The MAC in the* Response* message is generated using the group key and is used for detecting a forged* Response* message.  Figure 4 shows the exchange of* Hello* and* Response *messages among neighbor nodes.

When the node sending the* Hello* message receives a* Response* message from one of its neighbors, it verifies the MAC using its group key. If the verification succeeds, it adds the receiver's ID to its neighbor nodes list. It also stores the distance from the neighbor to the sink node, the energy consumption of the neighbor, and the secret key indexes of the neighbor in the list. If the neighbor is closer to the sink node than the current node, it can be selected by the current node as a next node to which a key dissemination message or an event report can be forwarded. In the proposed method, we define such a neighbor node as a candidate parent node (CPN) of the current node. The neighbor discovery phase and the following key dissemination phase are executed in every round.

In the key dissemination phase, each node except the CH updates its authentication key, encrypts the authentication key using one of its secret keys, and sends the authentication key to the CH. The CH collects the encrypted authentication keys from its member nodes and generates a key dissemination message. Equation (5) shows the format of the key dissemination message:

(5) {v1,i1,j1,{AKi1v1}SKj1, v2,i2,j2,{AKi2v2}SKj2,     ⋮ vn,in,jn,{AKinvn}SKjn}.

Each key dissemination message includes the node IDs (*v*_1_ − *v*_*n*_), the authentication key indexes (*i*_1_ − *i*_*n*_), the secret key indexes (*j*_1_ − *j*_*n*_) used for encryption of the authentication keys, and the encrypted authentication keys ({AK_*i*_1__^*v*_1_^}_SK_*j*_1___ − {AK_*i*_*n*__^*v*_*n*_^}_SK_*j*_*n*___). Any encryption algorithms, such as RC4 [25] and TEA [26], can be exploited for the encryption/decryption of authentication keys.

After creation of the key dissemination message, the CH selects a few nodes from among its CPNs and forwards the key dissemination message to them. The number of CPNs selected for the next-hop nodes at each node is determined by the system parameter BRANCH_FACTOR(BF). For example, if the BF is two, every node on the forwarding path selects two CPNs as the next-hop nodes.

The topology of a sensor network may change frequently for many reasons such as low duty cycle [27], energy depletion, or destruction of sensor nodes. Therefore, the value of BF should be larger than one so that the proposed method adapts to the dynamic topology changes of the network. As BF increases, the number of nodes to which authentication keys are distributed increases. However, if BF is too large, the number of different authentication keys stored at sensor nodes may decrease due to limited memory of the sensor nodes. For DEF, in [11], the authors selected the values (2–6) for BF. Therefore, we assumed that BF is two in our experiments, which are described in (Section 5—Experimental Results).

The CH and other forwarding nodes derive the fitness values of their CPNs to be selected as the next-hop nodes based on the evaluation function in

(6) $$\begin{array}{c} f\left(N\right)=\alpha_{P}\cdot \text{DK}\left(N\right)-\left(1-\alpha_{P}\right)\cdot \left\{\text{DI}\left(N\right)+\text{EC}\left(N\right)\right\}. \end{array}$$

**Figure 5:**
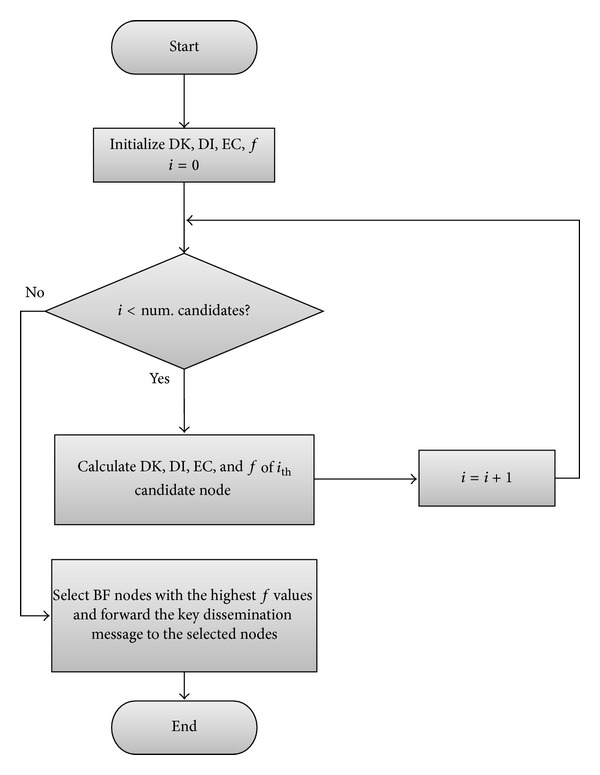
Internal operation of a node that receives a key dissemination message.

In (6), *N* is one of CPNs of the current node and DK(*N*) is the number of authentication keys that node *N* can obtain from the key dissemination message. The current node can derive the value of DK(*N*) by considering the secret key indexes of node *N* and the secret key indexes of the key dissemination message. DI(*N*) and EC(*N*) correspond to the distances from node *N* to the sink node and the energy consumption of node *N*, respectively. The parameters *α*_*P*_ and (1 − *α*_*P*_) represent the weight values for the terms. The current node computes the evaluation function for each CPN and selects BF nodes from among them to be the next-hop nodes. It then forwards the key dissemination message to the selected next-hop nodes.  Figure 5 illustrates the internal operation of a node that receives a key dissemination message.

**Figure 6:**
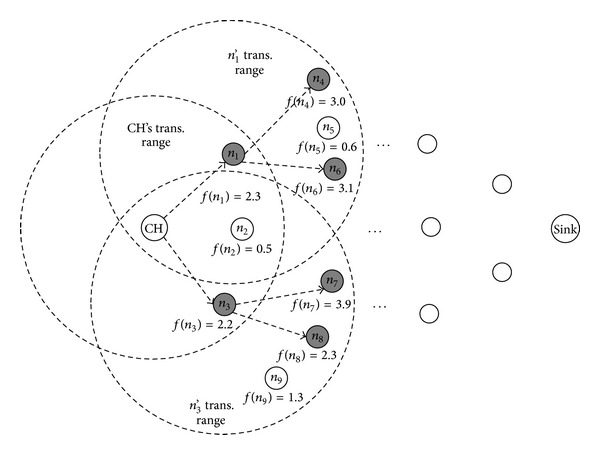
Key dissemination in the proposed method.

When a node receives a key dissemination message, it first initializes the values of the input parameters (DK, DI, and EC) and output variable (fitness value *f*) of a candidate node. It also initializes the number of iterations (*i*) to zero. If the number of iterations is less than the number of CPNs of the receiving node, it computes the DK, DI, and EC of the next CPN and derives the corresponding fitness value of the CPN. Then the receiving node increases the number of iterations by one. After computing fitness values for all the CPNs, the receiving node selects BF nodes with the highest fitness values among all the CPNs. Finally, it forwards the key dissemination message to the selected nodes.  Figure 6 illustrates the forwarding of the key dissemination message in the proposed method.

The gray circles in Figure 6 represent the nodes selected as the next-hop nodes by the nodes on the key-dissemination-message forwarding path. In the above example, the BF is two, and therefore each node selects two nodes with the highest fitness values from among its CPNs.

A node receiving a key dissemination message compares its secret key indexes with the secret key indexes in the key dissemination message. If there is a matching secret key, the node decrypts the corresponding authentication key and stores the authentication key with the corresponding node ID and the authentication key index in its memory. The maximum number of hops for each key dissemination message is limited by the system parameter time-to-live (TTL).

In the data dissemination phase, member nodes of each cluster generate sensing reports and send them to their CH periodically or when an event occurs. A sensing report is composed of the sensing data, the sensing node's ID, the authentication key index of the sensing node, and the MAC generated using the authentication key. The CH organizes the final report from the received sensing reports. The format of the final report is as follows:

(7) {SD,v1,i1,MAC(SD,AKi1v1), v2,i2,MAC(SD,AKi2v2),      ⋮ vk,ik,MAC(SD,AKikvk)}.

The final report contains the sensing data (SD), the IDs  (*v*_1_ − *v*_*k*_), and authentication key indexes (*i*_1_ − *i*_*k*_) of the sensing nodes. The CH selects from among its CPNs the next-hop node to which the final report will be forwarded:

(8) $$\begin{array}{c} f^{\prime }\left(N\right)=\alpha_{P}\cdot {\text{DK}}^{\prime }\left(N\right)-\left(1-\alpha_{P}\right)\cdot \left\{\text{DI}\left(N\right)+\text{EC}\left(N\right)\right\}. \end{array}$$

In (8), *N* is a CPN of the current node and *f*′(*N*) is the fitness value of node *N* to be selected as the next node to which the final report will be forwarded. DK′(*N*) is the number of authentication keys node *N* has previously obtained from the source cluster in the last key dissemination phase. DI(*N*) and EC(*N*) correspond to the distance from node *N* to the sink node and the energy consumption of node *N*, respectively. The parameter *α* is the weight value for DK′(*N*).

Each node on the forwarding path of the final report derives the fitness values of its CPNs and chooses the node with the maximum fitness value as the next-hop node on the forwarding path. Then, it forwards the final report to the selected node.

A node receiving the final report compares the authentication key indexes in its memory with the authentication key indexes in the final report. If there is a matching authentication key, the node verifies the corresponding MAC and forwards it to the next node only when the verification result is true. If the verification fails, the false report is detected and removed by the receiving node.

### 4.2. Computation Overhead of the Proposed Method

We assume that one of the cluster-based routing protocols is exploited in the proposed method. Therefore, only CHs evaluate fitness values of their CPNs and forward key dissemination messages or event reports to the selected next-hop nodes. According to sensor network applications, a CH may be a powerful device (e.g., with greater battery power, more capable CPU, and longer transmission range) or it can be a normal sensor node. The evaluation function shown in (6) is composed of primitive operators such as addition, subtraction, multiplication, and comparison.

Let us assume that *n*  is the number of authentication keys in each key dissemination message and *k* is the number of secret keys assigned to each node. Then, a CH performs *n*∗*k* comparison for computing DK(*N*) (i.e., the number of authentication keys that node *N* can obtain from the key dissemination message), one addition, two subtraction, and two multiplication operations for computing the fitness values of its CPNs. In addition to that, the CH sorts the list of CPNs based on their fitness values. The computational complexity of a sorting algorithm is (*nc*log⁡*nc*), where *nc* is the number of CPNs. However, in our proposed method the value of *nc* is small (between 1 and 5) and therefore the execution time is short. As a result, the computation overhead of the proposed method is practical for typical sensor nodes.

### 4.3. Impact of Node Density on Key Dissemination

We assumed, in (Section 4.1—Operation), that the node density is sufficient so that each node is associated with at least one CPN. Therefore, we need to analyze the relationship between the node density *d* and the number of CPNs *nc*.

If the transmission range of each node is *r*, then the area within the transmission range of the node should be *πr* ^2^. The number of neighbor nodes within the area, *n*, determines *nc*, which is the number of CPNs, probabilistically.

The probability *p* that a node does not have any candidate parent node can be calculated as follows:

(9) $$\begin{array}{c} p={\left(\frac{1}{2}\right)}^{n}. \end{array}$$

The node density *d* can be calculated as follows:

(10) $$\begin{array}{c} d=\frac{n}{\pi r^{2}}. \end{array}$$

From (9) and (10), we can derive the following equation:

(11) $$\begin{array}{c} p={\left(\frac{1}{2}\right)}^{d\pi r^{2}}. \end{array}$$

**Figure 7:**
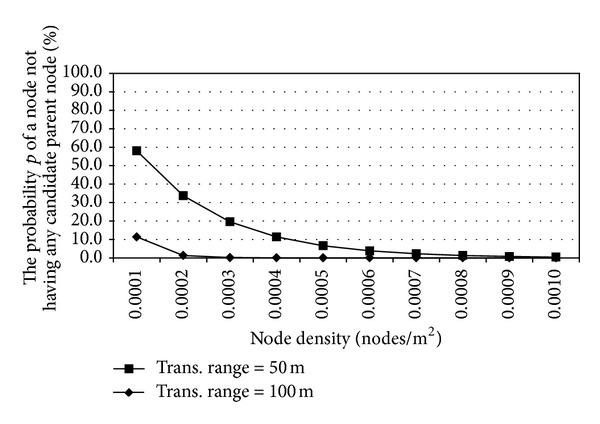
The probability of no candidate parent node versus the node density.

According to (11), we can then represent the relationship between the node density *d* and the probability *p* as shown in Figure 7.

We can see in Figure 7 that, as the node density *d* increases, the probability *p* that a node does not have any candidate parent node decreases. In addition, as the transmission range *r* of a node increases, the probability *p* also decreases. For example, when *r* = 50 and *d* = 0.0001, *p* = 58%, and when *r* = 58 and *d* = 0.005, *p* = 6.6%. In another case, when *r* = 100 and *d* = 0.0004, *p* is almost zero.

As we explained in (Section 4.2—Computation Overhead of the Proposed Method) only CHs can participate in the forwarding process of the key dissemination messages and event reports in the proposed method. That is, the number of CPNs for a given CH is actually the same as the number of neighboring CHs within its transmission range. Therefore, a clustering scheme should guarantee at least BF (i.e., a branch factor) neighboring CHs for each CH. If the number of neighboring CHs is less than BF, the number of CPNs of the CH becomes less than BF. As a result, the number of nodes to which the authentication keys in the source cluster are actually distributed is decreased. On the other hand, if the number of neighboring CHs is too large (e.g., 20), the computation overhead to perform the evaluation function shown in (6) increases.

For example, CHs are elected in LEACH [24] based on a probabilistic approach. Therefore, we can easily control *nc* (the number of CPNs for each CH) by choosing the desired percentage of CHs based on the total number of nodes and the field size. On the other hand, a node decides its role in the PebbleNet [23] based on its weight value and the weight values of its neighbor nodes. That is, for a given CH all the other nodes within its transmission range become ordinary nodes within the cluster. Therefore, it is hard to control the number of CHs in PebbleNet. Since we assume that we need at least BFneighboring CHs for a given CH, LEACH is more suitable for the proposed method compared to PebbleNet.

## 5. Experimental Results

In our experiments the size of the network field is 500  ×  500 m^2^ and includes 1,000 sensor nodes. Each cluster is organized by ten sensor nodes. The size of each cluster is 50 × 50 m^2^, and hence, there are 100 clusters in the field. The transmission range of each node is 50 m.

The GKP is composed of 100 secret keys. Each node is assigned five secret keys from the GKP. It can also store its own authentication key, and it can obtain at most ten authentication keys from other clusters for false report detection.

We assume that in our experiment the branch factor BF for forwarding the key dissemination message is two, both using GEAR and using the proposed method. Therefore, each node forwards the key dissemination message to at most two of its neighbors. The weight value of *α*_*G*_ for GEAR is 0.5, whereas the weight value of *α*_*P*_ for the proposed method varies from zero to one.

We assumed in the proposed method that every message (including the key dissemination message and the final event report) has the same length of 36 bytes, since TinyOS [28] uses packets of 36 bytes or less. The energy consumption for sending/receiving one byte is 16.25/12.5 *μ*J and the energy consumption for verifying a* MAC* in the final report is 75 *μ*J [29, 30].

Each final report contains five MACs, one of which is a false MAC when the report was forged by an attacker. The false traffic ratio (FTR) is the number of false reports divided by the total number of final reports generated and forwarded. We performed experiments for various FTR values (0–100%).

**Figure 8:**
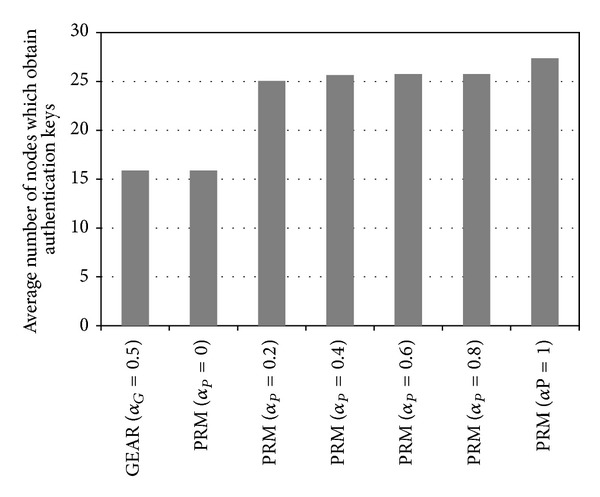
Average number of nodes that obtain authentication keys from a source cluster.

The proposed method selects the next nodes to which the key dissemination messages or final event reports are forwarded based on the secret key indexes of CPNs. Therefore, the proposed method can increase the number of nodes to which authentication keys in each cluster are distributed.  Figure 8 shows the average number of nodes that obtain authentication keys from a source cluster in GEAR and the proposed method (PRM).

**Figure 9:**
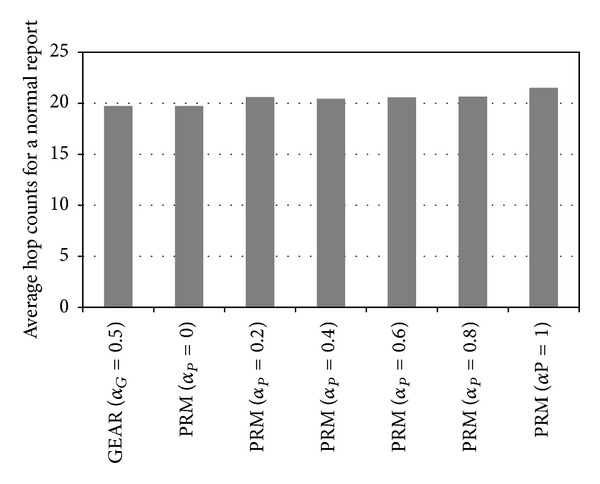
Average hop counts for a normal report.

As the value of *α*_*P*_ for the evaluation function of the proposed method increases, the priority of the DK(*N*) in (6) increases. Therefore, the number of nodes that obtain authentication keys increases as the value of *α*_*P*_ increases. For example, when *α*_*P*_ = 0.4, the number of nodes that obtain the authentication keys from a cluster in the proposed method is 162% (25.6) of the number in GEAR (15.9). The proposed method does not choose the shortest path from the source cluster to the sink node for final reports. Therefore, we need to measure the average hop counts that the normal or false report passes, to analyze the energy efficiency of the proposed method.  Figure 9 illustrates the average hop count per normal report in GEAR and in the proposed method.

**Figure 10:**
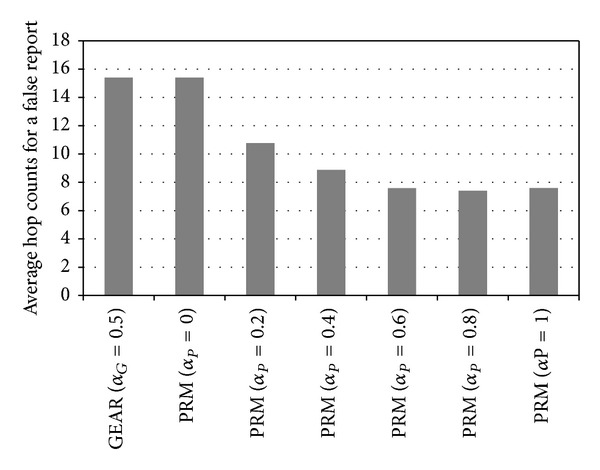
Average hop counts for a false report.

In the proposed method, the number of hops per normal report increases as *α*_*P*_ increases. To illustrate, when *α*_*P*_ = 0.2, the number of hops that a normal report passes in the proposed method is 104% of that in GEAR. In another case, even when *α*_*P*_ = 1, the number of hops in the proposed method is 109% of that of GEAR. Therefore, the proposed method has less than 10% more hops than GEAR.  Figure 10 shows the average hop count per false report in GEAR and in the proposed method.

**Figure 11:**
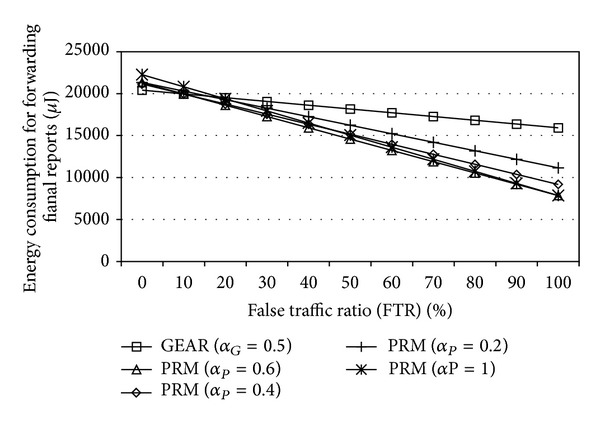
Energy consumption for forwarding final reports.

It is obvious that the proposed method reduces the average hop counts per false report significantly compared to GEAR. For example, when *α*_*P*_ = 0.2, the number of hops that a false report passes in the proposed method is 70% of that in GEAR. In another case, when *α*_*P*_ = 0.8, the number of hops that a false report passes in the proposed method is 48% of that in GEAR. Therefore, the proposed method significantly reduces the average hop count before a false report is detected. From the above results, we can derive the energy consumption for forwarding final reports for varying FTR values, as in Figure 11.

It is evident that GEAR consumes less energy for forwarding final reports than the proposed method when FTR < 10%. The reason for this is that the average hop count for a normal report in the proposed method is higher than in GEAR. However, when FTR ≥ 10%, the proposed method consumes less energy than GEAR since the average hop count for a false report in the proposed method is far less than GEAR. For example, when FTR = 50%, the energy consumption for forwarding final reports in the proposed method (*α*_*P*_ = 0.6) is 80% of that in GEAR.

**Figure 12:**
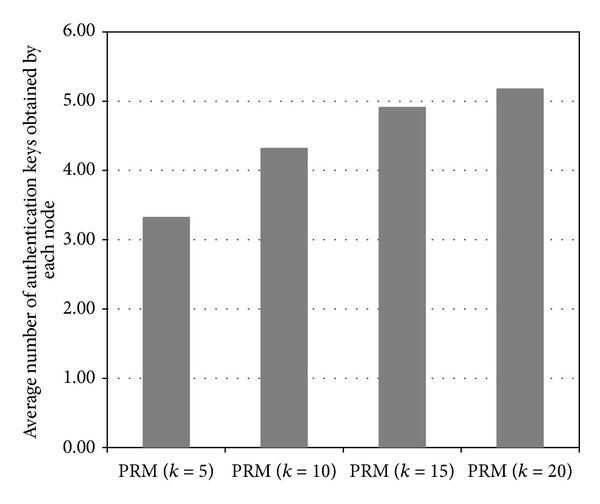
The number of authentication keys obtained by each node for different *k* values (*α*_*P*_ = 0.2).

The number of secret keys assigned to each node from the* GKP* is determined by the system parameter *k* in the proposed method. The value of *k* affects the performance of the proposed method in terms of (1) the number of authentication keys obtained from key dissemination messages by a node and (2) the average hop counts for a false report. Therefore, we observed the impact of *k*  on the performance of the proposed method based on the two performance measures through experiments, as follows.  Figure 12 shows the number of authentication keys obtained by each node for different *k* values.

**Figure 13:**
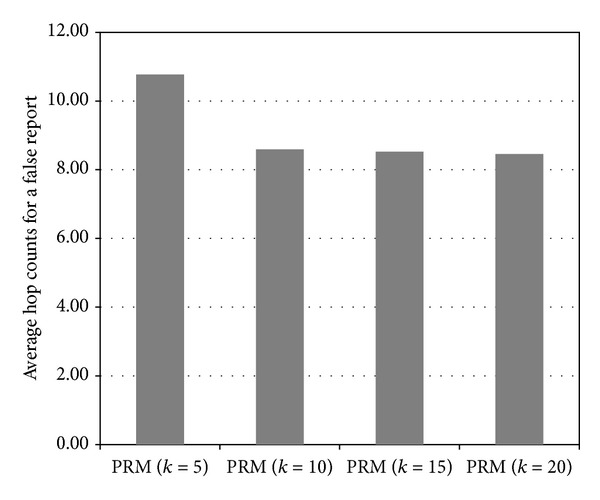
Average hop counts per false report for various *k* values (*α*_*P*_ = 0.2).

In the experiment, the total number of secret keys in the GKP is 100, and the maximum number of authentication keys that a node can obtain from other clusters (through key dissemination messages) is 10. We can see that the larger the value of *k*, the more the authentication keys a node can obtain from other clusters through key dissemination messages. When *k* is 5, 10, 15, or 20, a sensor node can, respectively, obtain on average 3.33, 4.33, 4.92, or 5.18 keys from other clusters. On the other hand, the degree of performance improvement decreases as *k* increases because the number of authentication keys that a sensor node can obtain from other clusters is limited by the fixed memory size of the nodes. Moreover, key dissemination messages are forwarded through the paths that are composed of nodes with high fitness values. Therefore, the authentication keys tend to be concentrated at some nodes, which leads to a lack of memory in the nodes.  Figure 13 shows the average hop counts for a false report for different *k* values.

As we have seen in Figure 12, as the value of *k* increases, the number of authentication keys a node obtains from other clusters increases. Therefore, the probability that a node detects a false report also increases and the average number of hops per false report decreases. When *k* is 5, 10, 15, or 20, the average hop counts for a false report is 10.78, 8.59, 8.52, and 8.46 hops, respectively, by the intermediated nodes on the forwarding path.

For the same reason as for the results shown in the Figure 12, the degree of performance improvement in terms of the average number of hops per false report is limited as *k* increases. In summary, as the number of secret keys assigned to each node from the GKP increases, the performance of the proposed method is improved. However, the degree of improvement is limited because of the memory constraints of nodes.

## 6. Conclusion

Authentication is an important security mechanism used for detecting forged messages in a sensor network. In the authentication key dissemination phase, the routing paths of the key dissemination messages strongly affect the number of nodes to which authentication keys are distributed. Here, we have proposed a routing method for key dissemination messages that increase the number of nodes to which authentication keys are actually distributed. The selection of next-hop nodes to which the key dissemination message is forwarded is based on the secret key indexes, the distance to the sink node, and the energy consumption of the CPNs. We have confirmed through experimentation that, in the proposed method, authentication keys in a cluster are distributed to an average of 50–70% more nodes than in GEAR. In addition, when FTR > 10%, the proposed method consumes 49–95% of the energy in GEAR for forwarding final reports. As a result, the proposed method can detect false reports earlier and so prolong the network lifetime.
